# Pre- and postpartum fear of childbirth and its predictors among rural women in China

**DOI:** 10.1186/s12884-024-06585-x

**Published:** 2024-05-30

**Authors:** Rong Xu, Jiarun Wang, Yuejie Li, Yujia Chen, Wei Zhang, Xinlong Pan, Zhijie Zou, Xiaoli Chen, Shuyuan Huang

**Affiliations:** 1 School of Nursing, Wuhan University, No. 115 Donghu Road, Wuhan, 430071 Hubei Province China; 2 Guoyang County People’s Hospital, Anhui, China; 3 Department of Obstetrics and Gynecology, Zhongnan Hospital of Wuhan University, Wuhan, China; 4 Department of Hematology, Zhongnan Hospital of Wuhan University, Wuhan, China; 5 NYU Rory Meyers College of Nursing, New York, USA

**Keywords:** Fear, Childbirth, Prenatal, Postpartum, Rural women

## Abstract

**Background:**

Fear of childbirth (FOC) can influence both maternal and child health. Research on FOC in China is scarce, especially on rural women. This study aimed to assess pre- and postpartum FOC and its predictors among Chinese rural women.

**Methods:**

This was a prospective correlation study. A total of 569 women completed the prenatal questionnaire in the third trimester, and 477 of them completed the postpartum questionnaire within three days after childbirth. Maternal socio-demographic information, clinical information, childbirth self-efficacy and prenatal and postpartum FOC were investigated. FOC was evaluated using the Wijma Childbirth Expectancy/ Experience Questionnaire (WDEQ). Descriptive, bivariate, multivariate linear regression analysis, univariate and multivariate logistic regression analyses were performed.

**Results:**

The mean pre- and postpartum FOC scores were 64.5 (standard deviation: 25.1) and 64.3 (standard deviation: 23.9), respectively, with 20.8% of women reporting severe fear before childbirth and 18.2% after childbirth. Multivariate linear regression analysis revealed predictors for higher levels of prenatal FOC including higher education level, nullipara, higher monthly household income, lower family support, and lower childbirth self-efficacy (*p* < 0.05) and the predictors for higher levels of postpartum FOC included unemployed status, lower childbirth self-efficacy, and higher prenatal FOC (*p* < 0.05). Multivariate logistic regression showed that higher childbirth self-efficacy reduced the likelihood of severe prenatal FOC (OR: 0.99, *p* < 0.001), while severe prenatal FOC increased the likelihood of severe postpartum FOC (OR: 3.57, *p* < 0.001).

**Conclusion:**

The rural women have high levels of FOC before and after childbirth, with approximately 20% experiencing severe FOC during both periods. Higher education level, nullipara, higher monthly household income, lower family support, and lower childbirth self-efficacy are predictors of heightened prenatal FOC. Unemployed status, lower childbirth self-efficacy, and higher prenatal FOC are predictors of heightened postpartum FOC. Notably, enhancing childbirth self-efficacy emerges as crucial in mitigating severe prenatal FOC, while severe prenatal FOC significantly increases the likelihood of severe postpartum FOC. The development of targeted intervention strategies for the above factors can help reduce women’s FOC level and improve their overall pregnancy and childbirth experience.

**Supplementary Information:**

The online version contains supplementary material available at 10.1186/s12884-024-06585-x.

## Background

Childbirth is a significant event for most women and is widely recognized as a transformative phase of motherhood involving physical, psychological, and social aspects [1]. Women may experience psychological distress and anxiety when faced with labor, which can lead to fear of childbirth (FOC). FOC is a state of uncertainty or anxiety that can occur before, during, or after childbirth; can arise from thoughts about the impending labor; or can be influenced by the fearful reactions of others to the labor process [2]. FOC can range from normal worries and fears to severe fears [3]. The prevalence of FOC varies widely between countries and regions, ranging from 3.7 to 43%, with a recent upward trend [4–7]. Meta-analyses suggest that the global prevalence of severe FOC is approximately 14% [5, 6]. Despite the availability of safe childbirth care in numerous countries, childbirth can be a heavy burden for some women.

FOC can have severe consequences for maternal and infant health, including increased requests for cesarean sections, prolonged labor, postpartum hemorrhage, fetal distress, intrauterine growth retardation, preterm birth, impaired mother–infant bonding, and breastfeeding difficulties [8–10]. FOC can reduce women’s quality of life and satisfaction with childbirth, increase their stress, anxiety, and likelihood of postpartum depression and posttraumatic stress symptoms, and affect family relationships, future fertility intentions, and choice of mode of childbirth [11–14]. Therefore, it is essential to identify and assess FOC during and after childbirth to promote the well-being of mothers and their children.

Various factors, such as demographic, obstetric, and psychological factors, can influence FOC. Socioeconomic status, education, social support, childbirth self-efficacy, anxiety, depression, and abuse history are common factors for FOC [15–19]. However, several factors remain controversial. Some studies found that high prenatal FOC was associated with primiparity [3, 6, 20], while two studies found no significant difference [21, 22]. One study showed that illiterate women were more likely to have high prenatal FOC than educated women [8]. In contrast, other studies have shown that college-educated women are more likely to have high prenatal FOC than less-educated women [23, 24]. These differences highlight the need for further exploration and research to better understand the factors influencing prenatal FOC. Education level, employment status, and prenatal FOC may be related to postpartum FOC [8, 25]. However, there are few studies on postpartum FOC and its factors, and further exploration of the influencing factors of postnatal FOC is needed.

FOC can be assessed using various methods, including interviews, questionnaires, and physiological measures. One of the most widely used questionnaires is the Wijma Delivery Expectations/Experience Questionnaire (WDEQ), which measures prenatal FOC by considering women’s expectations of childbirth and postpartum FOC by considering their childbirth experience [26, 27].

Although there have been some studies on FOC in China recently [15, 23, 28], most have focused mainly on the prenatal period. There are few studies on the postpartum period, and none have examined both periods. In addition, research on women’s FOC in rural areas has not been conducted. According to China’s seventh national census in 2020, approximately 500 million people live in rural areas, accounting for 36.11% of China’s population. Maternal health care in rural China remains a significant challenge due to the lack of medical resources and a comprehensive prenatal care system. The rural population in China accounts for a large proportion of the population and has unique social and cultural characteristics. To date, no study has evaluated the FOC of rural mothers in China before and after childbirth.

Therefore, it is important to assess FOC and its associated factors in the prenatal and postpartum periods among rural Chinese women. This study aimed to (1) investigate the status of pre- and postpartum FOC among Chinese rural women; (2) investigate the predictors of pre-and postpartum FOC among Chinese rural women. By exploring these findings, we hope better to understand the status of FOC among Chinese rural women and identify its predictors.

## Methods

### Study design and settings

The study used a prospective correlational design. Participants were recruited from April to December 2020 from the obstetrics ward of the People’s Hospital of Guoyang County, Anhui Province, China, using convenience sampling. According to the 2020 National Census of China, the rural population of Guoyang County accounted for 61.74% of the total population. Guoyang County People’s Hospital is a Grade 2 A comprehensive hospital and the largest provider of women’s health services in Guoyang County.

### Sample size

We used the following formula to calculate the sample size [29].

$$N={\left(\frac{{Z}_{{\alpha }/2} }{{\delta }}\right)}^{2}\pi (1-\pi )$$

π indicated the detection prevalence of severe FOC. By reviewing the literature, a meta-analysis by Nilsson et al. revealed that the global prevalence of severe FOC is 14% [5], thus π in this formula in our study is 14%. The significance level was ⍺= 0.05, and a tolerance error δ = 0.04 was set for the calculation. The estimated sample size required was 289 women, and considering a 20% dropout rate at each follow-up (1 follow-up in total), a minimum of 347 participants were required to be included in the study baseline.

### Study participants

Participants in this study were women who received pre- and postpartum care in this hospital. Potential participants were approached by the researchers or referred by midwives to participate in the study. We included women who met the following criteria: (a) were at least 18 years old; (b) had rural household registration; (c) were able to communicate in written and spoken Chinese; (d) had singleton pregnancies in the third trimester; and (e) planned to give birth at our research hospital. The exclusion criteria were as follows: (a) had mental illness; (b) had chronic diseases before this pregnancy; (c) individuals who had a stillbirth were excluded from the postpartum period.

### Data collection

Data collection occurred in two stages. During the third trimester, eligible pregnant women who agreed to participate were required to complete the prenatal questionnaire on paper. Within three days after childbirth, the women were followed up and asked to complete the postpartum questionnaire on paper.

### Measurements

#### Sociodemographic and clinical information

Demographic and clinical information collected in the third trimester included age, education level, marital status, employment status, parity, planning pregnancy, family support, preferred mode of childbirth, prenatal examinations, monthly household income, gestational week, abortion history, complications of pregnancy, and whether have siblings. The actual mode of childbirth and gestational week at birth were collected via the questionnaire within three days after childbirth.

#### Childbirth self-efficacy

Childbirth self-efficacy was measured in the third trimester of pregnancy using the short form of 32-item Chinese Childbirth Self-Efficacy Inventory (CBSEI-C32). The CBSEI-C32 is a 32-item self-reported questionnaire with a 10-point Likert scale ranging from 1 (not at all helpful/sure) to 10 (very helpful/completely sure). The total score ranges from 32 to 320, with higher scores indicating greater childbirth self-efficacy [30]. The CBSEI-C32 was developed by IP et al. based on the CBSEI which was designed and developed by Lowe [30, 31]. The original CBSEI-C32 reported a Cronbach’s alpha of 0.96 [32]. In this study, the Cronbach’s alpha of the CBSEI was 0.90.

#### Pre- and postpartum FOC

FOC were measured using the Chinese version of the Wijma Delivery Expectancy/ Experience Questionnaire (WDEQ) version A and B [27]. Prenatal FOC were assessed using version A (WDEQ -A), and postpartum FOC were assessed using version B (WDEQ -B). The WDEQ is a 33-item self-reported questionnaire with a 6-point Likert scale ranging from ranging from 0 (not at all) to 5 (extremely), with a total score from 0 to 165 [27]. Scores ≤ 37, 38 to 65, 66 to 84, and ≥ 85 represent low, moderate, high, and severe fear, respectively [27]. The WDEQ was originally designed and developed by Swedish academic Klass Wijma [27]. Liu et al. translated the WDEQ into Chinese and and tested its validity [33, 34]. The Cronbach’s alpha for Chinese version of WDEQ -A and WDEQ -B were 0.93 and 0.92, respectively [33]. In this study, the Cronbach’s alphas of the WDEQ-A and WDEQ-B were 0.78 and 0.74, respectively.

### Statistical analysis

The data were analyzed using the Statistical Package for the Social Sciences (SPSS) version 25. Descriptive statistics summarized participant characteristics, including percentages, frequencies, means, and standard deviations (SD). For bivariate analyses, independent samples t-tests (employment status, marital status, parity, family support, planning pregnancy, preferred mode of childbirth, actual mode of childbirth, gestational week, gestational week at birth, complications of pregnancy, abortion history, whether have siblings and total FOC score), one-way ANOVA (prenatal examinations, education level, monthly household income and total FOC score) and Pearson correlation analysis (age, childbirth self-efficacy and total FOC score) were used for continuous variables. Paired t-test was used to test whether the means between prenatal and postpartum FOC scores were significantly different. The Wilcoxon rank-sum test was used to conduct a between-group paired test of the levels of pre- and postpartum FOC. The generalized linear regression analysis included variables with *P* < 0.2 in the univariate analysis for further analysis. In addition, univariate and multivariate logistic regression was used to analyze factors related with severe FOC (WDEQ scores ≥ 85). Variables with *p* < 0.2 in the univariate logistic regression analysis were included in the multivariate logistic regression analysis. Results are expressed as odd ratios (OR) and 95% confidence intervals (CI). All differences were tested using two-tailed tests, with the significance level set at *P* < 0.05.

## Results

**Fig. 1:**
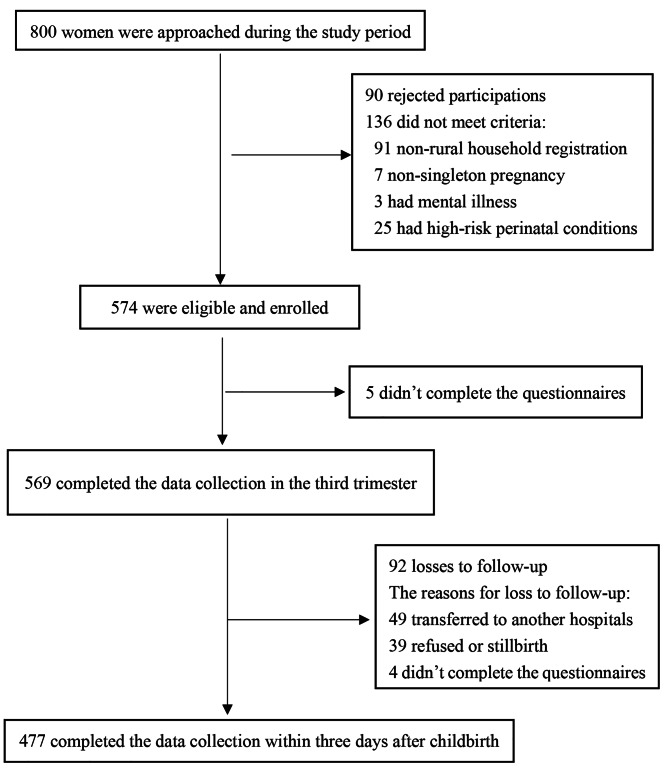
Flow chart of the study

Of the 800 women contacted, 710 expressed interests in the study and were screened, and 574 were eligible and enrolled. Ultimately, 569 completed the third trimester data collection, and 477 completed the two-stage data collection (Fig. 1).

The mean age of 569 participants was 26.8 (SD = 5.0) years (Table 1). The majority of the participants had a junior high school education or less (54.5%), were employed (51.7%), and came from low-income families (69.6%), which are below the Chinese well-off level (with a monthly household income of less than 6,667 RMB). More than half of the women reported that their current pregnancy was unplanned (52.5%). More than half were multiparas (51.82%). Most (85.1%) had no pregnancy complications and many (83.8%) said they had high family support. Of the 477 postpartum participants followed, the vast majority (96%) had full-term births and normal vaginal births (87.4%). The average score of childbirth self-efficacy was 235.8 (SD = 69.0). More details can be found in Table 1.

**Table 1:** The mean score of women’s fear of childbirth based on sociodemographic and clinical characteristics

Characteristics	*n* (%)/Mean (SD)	WDEQ-A (*N* = 569)		*n* (%)	WDEQ-B (*N* = 477)	
		Mean (SD)	*P*		Mean (SD)	*P*
Employment status^++^			0.298 ^a^			**0.005** ^a^
Employed	289 (51.7)	62.7 (25.4)		239 (50.1)	61.2 (24.7)	
Unemployed	275 (48.3)	64.9 (25.3)		235 (49.3)	67.3 (22.8)	
Marital Status			0.661 ^a^			0.579 ^a^
Married	558 (98.1)	63.7 (25.4)		467 (97.9)	64.3 (24.0)	
Other	11 (1.9)	60.3 (25.3)		10 (2.1)	60.1 (22.5)	
Parity			**< 0.001** ^a^			**0.003** ^a^
Nullipara	274 (48.2)	67.6 (23.1)		230 (48.2)	67.7 (22.3)	
Multipara	295 (51.8)	59.9 (26.8)		247 (51.8)	61.1 (25.0)	
Planning Pregnancy^++^			0.920 ^a^			0.909 ^a^
Planned	268 (47.1)	63.7 (26.1)		220 (46.1)	64.4 (24.1)	
Unplanned	299 (52.5)	63.5 (24.9)		256 (53.7)	64.1 (23.9)	
Family support^++^			**0.011** ^a^			0.153 ^a^
High	477 (83.8)	62.7 (25.3)		397 (83.3)	63.9 (24.1)	
Low	66 (11.6)	71.2 (24.0)		56 (11.7)	68.8 (20.6)	
Preferred MOC			0.765 ^a^			
Normal vaginal birth	516 (90.7)	63.7 (25.4)		∼	∼	∼
Cesarean childbirth	53 (9.3)	62.6 (25.2)				
Actual MOC						0.462 ^a^
Normal vaginal birth	∼	∼	∼	417 (87.4)	64.0 (23.9)	
Cesarean childbirth				60 (12.6)	66.4 (24.4)	
Gestational week (week)			**0.032** ^a^			
< 37	34 (6.0)	72.7 (24.4)		∼	∼	∼
37–42	535 (94.0)	63.0 (25.4)				
Gestational week at birth (week)						0.902 ^a^
< 37	∼	∼	∼	19 (4.0)	63.6 (24.1)	
37–42				458 (96.0)	64.3 (24.2)	
Complications of pregnancy			0.806 ^a^			0.920 ^a^
No	484 (85.1)	63.5 (25.3)		409 (85.7)	64.3 (23.5)	
Yes	85 (14.9)	64.2 (25.8)		68 (14.3)	64.0 (26.7)	
Abortion history			0.347 ^a^	274 (57.4)	65.3 (23.0)	0.290 ^a^
No	338 (59.4)	64.4 (24.5)		203 (42.6)	62.9 (25.2)	
Yes	231 (40.6)	62.4 (26.6)				
Whether have siblings			0.625 ^a^			0.937 ^a^
No	556 (97.7)	63.4 (25.4)		466 (97.7)	64.2 (24.1)	
Yes	13 (2.3)	67.0 (26.5)		11 (2.3)	64.8 (18.2)	
Prenatal examinations^++^			**0.034** ^b^			0.533 ^b^
Often	461 (81.0)	62.3 (25.9)		380 (79.7)	63.6 (23.9)	
Sometimes	60 (10.5)	67.9 (24.9)		54 (11.3)	67.4 (27.0)	
Never	44 (7.7)	71.0 (18.3)		39 (8.2)	64.9 (20.2)	
Education level^++^			0.188 ^b^			0.953 ^b^
Junior college or above	161 (26.9)	65.8 (26.6)		255 (47.2)	64.0 (23.2)	
Senior high school	94 (16.5)	66.1 (20.0)		84 (17.6)	64.9 (23.1)	
Junior high school or below	310 (54.5)	62.0 (25.8)		136 (28.5)	64.5 (26.0)	
Monthly household income (RMB)			0.068 ^b^			0.100 ^b^
< 3000	86 (15.1)	57.8 (29.2)		74 (15.5)	60.6 (25.5)	
3000–5000	253 (44.5)	65.1 (24.4)		215 (45.1)	64.7 (23.6)	
>5000	230 (40.4)	64.1 (24.7)		188 (39.4)	65.2 (23.7)	
Age (years)	26.8 (5.0)		**< 0.001** ^c^	26.7 (5.1)		**0.025** ^c^
Childbirth self-efficacy	235.8 (69.0)		**0.002** ^c^	234.4 (68.8)		**< 0.001** ^c^

Preferred MOC: Preferred Mode of Childbirth; Actual MOC: Actual Mode of Childbirth; ^a^ Independent sample t-test; ^b^ one-way ANOVA; ^c^ Pearson’s correlation analysis; ^++^ The variable data has missing values; SD: Standard Deviation

The mean score of prenatal FOC among 569 participants was 63.6 (SD = 25.4). Among the 477 women who participated in both periods, the mean pre- and postpartum FOC scores were 64.5(SD = 25.1) and 64.3(SD = 23.9), respectively (Supplementary Table 1). Among the 477 participants, there was a significant correlation between pre- and postpartum FOC scores (*p* < 0.001), but there was no significant difference in mean scores between the two periods (*p* = 0.808). There was no significant difference in the severity of FOC between the two periods (*p* = 0.247) (Supplementary Table 1). About 8% of women (*n* = 39) suffered severe FOC (scores ≥ 85) in both the prenatal and postpartum periods. 69% (*n* = 330) of women did not suffer severe FOC in either the prenatal or postpartum period. 13% of women (*n* = 60) who suffered prenatal severe FOC were not identified in the severe FOC group in the postpartum period. 10% of women (*n* = 48) had postpartum severe FOC and were not identified in the severe FOC group during the prenatal period. The results of the chi-square test indicate that the distribution of severe FOC before and after childbirth are statistically significant (*P* < 0.001) (Table 2).

**Table 2:** Comparison of severe fear of childbirth identification in pre- and postpartum Periods (*N* = 477)

	Severe postpartum FOC	Non-severe postpartum FOC	χ^2^ test	
	*n* (%)	*n* (%)	χ^2^	*p*
**Severe prenatal FOC**	39 (8.2)	60 (12.6)	37.49	**< 0.001**
**Non-severe prenatal FOC**	48 (10.1)	330 (69.2)		

The results of the multivariate linear regression analysis in Table 3 show that junior college education or above (B = 4.3, *P* = 0.044), monthly household income greater than 3000 RMB (B = 6.9, *P* = 0.011), receiving lower social support (B = 5.8, *P* = 0.040), nullipara (B = 5.8, *P* = 0.004), and low childbirth self-efficacy (B = -0.2, *P* < 0.001) can predict higher prenatal FOC score. Higher postpartum FOC score can be predicted by unemployed status (B = 4.2, *P* = 0.022), lower childbirth self-efficacy (B = -0.1, *P* = 0.009), and higher prenatal FOC score (B = 0.5, *P* < 0.001).

**Table 3:** Multivariate linear regression analysis of factors related with pre- and postpartum fear of childbirth

Variable	B	SE	95% CI for the B		*P*
			Lower	Upper	
**Prenatal period** (***N*** = **569**)					
Constant	94.5	7.0	80.8	108.3	< 0.001
Education level (ref: Junior high school or below)					
High School	2.3	2.6	-2.8	7.4	0.378
Junior college or above	4.3	2.1	0.1	8.4	**0.044**
Parity (ref: Multipara)					
Nullipara	5.8	2.0	1.8	9.7	**0.004**
Gestational week (ref: 37–42)					
<37	3.0	3.9	-4.5	10.6	0.434
Family support (ref: High)					
Low	5.8	2.8	0.3	11.4	**0.040**
Prenatal examinations (ref: Often)					
Sometimes	1.6	3.0	-4.4	7.5	0.611
Never	5.5	3.4	-1.2	12.2	0.107
Monthly household income (RMB) (ref:<3000)					
3000–5000	6.9	2.7	1.6	12.2	**0.011**
>5000	6.0	2.8	0.5	11.4	**0.031**
Age (years)	-0.1	0.2	-0.4	0.4	0.882
Childbirth self-efficacy (scores)	-0.2	0.1	-0.2	-0.1	**< 0.001**
**Postpartum period** (***N*** = **477**)					
Constant	39.5	7.9	23.9	55.1	< 0.001
Employment status (ref: Employed)					
Unemployed	4.2	1.9	0.6	7.8	**0.022**
Parity (ref: Multipara)					
Nullipara	3.2	2.1	-0.8	7.2	0.116
Family support (ref: High)					
Low	1.2	2.8	-4.3	6.8	0.660
Monthly household income (RMB) (ref: <3000)					
3000–5000	2.3	2.7	-3.0	7.6	0.394
>5000	3.2	2.8	-2.2	8.6	0.249
Age (years)	-0.1	0.2	-0.4	0.4	0.914
Childbirth self-efficacy (scores)	-0.1	0.1	-0.0	-0.1	**0.009**
WDEQ-A (scores)	0.5	0.1	0.4	0.5	**< 0.001**

B: Regression coefficient; SE: Standard Error

The results of multivariate logistic regression in Table 4 showed that women with higher childbirth self-efficacy (OR = 0.99, *p* < 0.001) were less likely to experience severe prenatal FOC (WDEQ-A scores ≥ 85). Women who had severe prenatal FOC (OR = 3.57, *p* < 0.001) were more likely to experience severe postpartum FOC (WDEQ-B scores ≥ 85).

**Table 4:** Univariate and multivariate logistic regression analysis of the related factors of severe fear of childbirth before and after childbirth

Variable	n (%)	Severe prenatal FOC (*n* = 111)		n (%)	Severe postpartum FOC (*n* = 87)	
		Univariate OR (95% CI), p	Multivariate OR (95% CI), p		Univariate OR (95% CI), p	Multivariate OR (95% CI), p
Employment status^++^						
Unemployed	62 (55.9)	1.43(0.94,2.17),0.096	1.54(0.97,2.44),0.069	50 (57.5)	1.52 (0.95,2.44),0.080	1.50 (0.89,2.52),0.125
Employed	49 (44.1)	Ref.	Ref.	36 (41.4)	Ref.	Ref.
Marital Status						
Other	1 (0.9)	0.41 (0.05 ∼ 3.21),0.394	∼	2 (2.3)	1.12 (0.23 ∼ 5.39),0.884	∼
Married	110 (99.1)	Ref.		85 (97.7)	Ref.	
Parity						
Nullipara	59 (53.2)	1.28 (0.85 ∼ 1.94),0.241	∼	50 (57.5)	1.58 (0.99 ∼ 2.52),0.057	1.41 (0.83 ∼ 2.41),0.206
Multipara	52 (46.8)	Ref.		37 (42.5)	Ref.	Ref.
Planning Pregnancy^++^						
Unplanned	56 (50.5)	0.89 (0.59 ∼ 1.35),0.591	∼	42 (48.3)	0.76 (0.48 ∼ 2.22),0.255	∼
Planned	55 (49.5)	Ref.		45 (51.7)	Ref.	
Family support^++^						
Low	16 (14.4)	1.38 (0.75 ∼ 2.53),0.303	∼	9 (10.3)	0.85 (0.40 ∼ 1.81),0.674	∼
High	90 (81.1)	Ref.		73 (83.9)	Ref.	
Preferred MOC						
Cesarean childbirth	9 (8.1)	0.83 (0.39 ∼ 1.76),0.626	∼	∼	∼	∼
Normal vaginal birth	102 (91.9)	Ref.				
Actual MOC						
Cesarean childbirth	∼	∼	∼	15 (17.2)	1.60 (0.85 ∼ 3.02),0.150	1.84 (0.92 ∼ 3.67),0.083
Normal vaginal birth				72 (82.8)	Ref.	Ref.
Gestational week						
< 37	10 (9.0)	1.79 (0.83 ∼ 3.86),0.138	1.24 (0.53 ∼ 2.91),0.626	∼	∼	∼
37–42	101 (91.0)	Ref.	Ref.			
Gestational week at birth						
< 37	∼	∼	∼	4 (4.6)	0.81 (0.27 ∼ 2.40),0.699	∼
37–42				83 (95.4)	Ref.	
Complications of pregnancy						
Yes	17 (15.3)	1.06 (0.59 ∼ 1.88),0.855	∼	12 (13.8)	0.95 (0.49 ∼ 1.87),0.891	∼
No	94 (84.7)	Ref.		75 (86.2)	Ref.	
Abortion history						
Yes	46 (41.4)	1.04 (0.69 ∼ 1.59),0.840	∼	35 (40.2)	0.89 (0.55 ∼ 1.43),0.627	∼
No	65 (58.6)	Ref.		52 (59.8)	Ref.	
Whether have siblings						
No	107 (96.4)	0.54 (0.16 ∼ 1.78),0.307	∼	85 (97.7)	1.00 (0.21 ∼ 4.73),0.996	∼
Yes	4 (3.6)	Ref.		2 (2.3)	Ref.	
Age(years)						
< 25	45 (40.5)	1.43(0.93 ∼ 2.19),0.102	1.31(0.83 ∼ 2.07),0.246	38 (43.7)	1.53 (0.96 ∼ 2.46),0.077	1.26 (0.73 ∼ 2.17),0.406
≥ 25	66 (59.5)	Ref.	Ref.	49 (56.3)	Ref.	Ref.
Education level^++^		0.288	0.051		0.223	0.236
Junior college or above	38 (34.3)	1.40(0.88 ∼ 2.23),0.155	1.75(1.05 ∼ 2.94),0.033	31 (35.6)	1.59 (0.94 ∼ 2.68),0.084	1.65(0.92 ∼ 2.95),0.092
Senior high school	16 (14.4)	0.93 (0.51 ∼ 1.71),0.817	0.84 (0.44 ∼ 1.62),0.610	16 (18.4)	1.27 (0.67 ∼ 2.40)0.0.473	1.30 (0.66 ∼ 2.60),0.449
Junior high school or below	56 (50.5)	Ref.	Ref.	40 (46.0)	Ref.	Ref.
Prenatal examinations ^++^		0.473	∼		0.649	∼
Never	9 (8.1)	1.14 (0.53 ∼ 2.46),0.743		6 (6.9)	0.85 (0.34 ∼ 2.11)	
Sometimes	15 (13.5)	1.48 (0.79 ∼ 2.77),0.227		12 (13.8)	1.34 (0.67 ∼ 2.67)	
Often	85 (76.6)	Ref.		67 (77.0)	Ref.	
Monthly household income (RMB)		0.473	∼		0.662	∼
>5000	40 (36.0)	0.92 (0.49 ∼ 1.75),0.802		37 (42.5)	1.40 (0.67 ∼ 2.93),0.366	
3000–5000	55 (49.5)	1.22 (0.65 ∼ 2.26),0.538		39 (44.8)	1.27 (0.61 ∼ 2.63),0.521	
< 3000	16 (14.4)	Ref.		11 (12.6)	Ref.	
Childbirth self-efficacy (scores)						
Mean (SD)	193.7 (74.8)	0.99 (0.98 ∼ 1.00),<0.001	0.99 (0.98 ∼ 1.00),**<0.001**	240.3 (66.1)	0.99 (0.99 ∼ 1.00),<0.001	1.00 (0.99 ∼ 1.00),0.056
WDEQ-A (scores)						
≥ 85	∼	∼	∼	39 (44.8)	4.47 (2.70 ∼ 7.40),<0.001	3.57 (2.06 ∼ 6.20),**<0.001**
< 85				48 (55.2)	Ref.	Ref.

Preferred MOC: Preferred Mode of Childbirth; Actual MOC: Actual Mode of Childbirth; ^++^ The variable data has missing values; SD: Standard Deviation

## Discussion

This study aimed to examine the status of pre- and postpartum FOC and its predictors among rural Chinese women. Our study found that rural women have high levels of pre- and postpartum FOC. Specifically, approximately one in five women reported severe FOC during both the prenatal and postpartum periods. The study revealed that higher education level, nullipara, higher monthly household income, lower family support, and lower childbirth self-efficacy are predictors of higher levels of prenatal FOC. Unemployed status, lower childbirth self-efficacy, and higher prenatal FOC are predictors of higher levels of postpartum FOC. In addition, women were more likely to experience severe FOC during the prenatal period if they had lower childbirth self-efficacy. Women who had severe prenatal FOC were more likely to experience severe postpartum FOC.

The results of our study showed that women in rural areas had significantly high level of prenatal FOC, which exceeded the levels found in relevant study in Canada [35] and were similar to those found in Iran [36] and Hong Kong, China [37]. Our study shows that more than half of the participants had relatively high prenatal FOC (WDEQ-A scores ≥ 66), which is higher than in some studies in Australia (26%), Portugal (28%) and Sweden (26%) [38–40]. It is similar to the study in Egypt (55.33%) [5]. Our findings indicate that approximately a fifth of rural women who participated in the study experienced severe FOC (WDEQ scores ≥ 85). This prevalence of prenatal severe FOC in Chinese rural women is greater than that in developed countries such as Canada, Sweden, Norway, Denmark and the Netherlands, where the prevalence of severe FOC ranges from 6.3 to 14.8% [5, 6]. The prevalence of severe prenatal FOC is higher or similar to that of some developing countries, such as Turkey, Kenya, Thailand, and Iran, where the prevalence of severe FOC ranges from 0.7 to 22.7% [41]. Furthermore, the prevalence of severe prenatal FOC was higher in rural women than in urban women in China, where the prevalence of FOC was 10.5% [34]. This may be due to the inadequacy of the medical resources in the rural areas. Compared with developed countries and Chinese urban areas, rural areas have fewer medical facilities and professionals, so pregnant women may not receive timely and necessary childbirth-related health education during pregnancy, which may affect their FOC levels. In addition, the larger gestational age of our participants (mean = 39.13 weeks) may be related to these issues. Some studies have found that the closer women are to childbirth, the higher their FOC scores [7, 42].

Our study found that rural women also had a high level of postpartum FOC, which was significantly higher than the Italian study [43]and similar to the Hong Kong, China study [44]. Our study showed the prevalence of severe postpartum FOC of nearly 20% among Chinese rural women. This is similar to a survey in Hong Kong, China (21.3%) [44]. More than half of the women in our study continued to have relatively high FOC (W-DEQ-B score ≥ 66) after childbirth. There was a small decrease in the percentage of women who exhibited high FOC compared to the prenatal period. This contradicts Khwepeya et al.‘s finding that high FOC decreases significantly after childbirth [8]. The reason for the high level of FOC in our study after childbirth may be due to the earlier collection of our postnatal data compared to the study by Khwepeya et al. Negative emotions during labor may affect postpartum FOC. A longitudinal study showed that adverse childbirth experiences are significantly associated with postpartum FOC [22]. To address this, healthcare institutions can provide childbirth education to reduce fear, and the government can invest in rural medical resources to ensure high-quality childbirth services for rural women.

Our findings showed that in rural areas, a subset of women experiences a significant shift in FOC status between the pre- and postpartum periods. Approximately 10% of women suffer from severe FOC during the postpartum period, whereas they were not identified as part of the severe FOC group during the prenatal period. A survey underscores the important influence of previous negative childbirth experiences on postpartum FOC [25]. Another study found that multiparous women with positive childbirth experiences have lower rates of postpartum FOC [45]. Since postpartum FOC is measured by considering a woman’s childbirth experience [27]. The results of our study show that some rural women have a poor childbirth experience, indicating a need for improvement in delivery care in rural healthcare facilities. Approximately 13% of women experiencing severe FOC during pregnancy were not identified as part of the severe FOC group during the postpartum period. This may reflect individual fluctuations in FOC levels during the perinatal period, warranting further research to understand the reasons and implications of such changes. About 8% of women experienced severe FOC both pre- and postpartum periods, indicating that this small subset may require ongoing attention and support to manage their fear emotions, which healthcare professionals should identify early. Additionally, while the majority of women (approximately 69%) did not experience severe FOC before and after childbirth, which is positive, it is also important to note that these women may experience other forms of anxiety or stress during pregnancy.

Our research showed that higher levels of prenatal FOC can be predicted by lower family support, nullipara, higher education level, higher monthly household income and lower childbirth self-efficacy. Several studies also found that nullipara [3, 6, 8, 20], low family support [15, 46], and low childbirth self-efficacy [47, 48] are predictors of higher levels of prenatal FOC. But the results of our study on education level [8] and relatively high income [20, 49] are inconsistent with some studies. These different findings reflect the complex and contradictory effects of socioeconomic status on rural Chinese women’s FOC. One study showed that lack of control during labor led to increased fear [50]. Well-educated women, in general, are used to planning and controlling their lives, which may be one reason why highly educated pregnant women experience higher levels of FOC. While higher income is associated with higher prenatal FOC, this may be related to the fact that pregnant women with higher incomes may have more resources and opportunities to pay attention to the risks and uncertainties of childbirth. Health professionals should identify high level of prenatal FOC early, based on education level, parity, and monthly household income. Modifiable factors such as childbirth self-efficacy and family support should also be identified, and interventions should be implemented to ensure that women have a positive childbirth expectancy.

We found that childbirth self-efficacy is not only an important predictor of higher levels of prenatal FOC, but also plays a critical role in reducing the likelihood of severe prenatal FOC. Self-efficacy refers to an individual’s belief in his or her ability to perform a behavior and achieve a desired outcome [31, 51]. Women with low childbirth self-efficacy may struggle to find motivation to cope with labor and childbirth, perceiving it as a difficult task [23]. Healthcare providers can increase women’s childbirth self-efficacy by providing knowledge about childbirth, pain management techniques, and breathing exercises, as well as sharing positive experiences and offering encouragement.

We found that higher levels of postpartum FOC can be predicted by unemployed status, childbirth self-efficacy and prenatal FOC. One study also found that unemployed status [8] is a predictor of higher levels of postpartum FOC. Previous studies have indicated that postpartum FOC level tend to increase with higher level of prenatal FOC [52, 53]. Health professionals can identify women with high levels of postpartum FOC early based on their employment status and high level of prenatal FOC, and promote women’s positive childbirth experience by improving their childbirth self-efficacy.

Our findings revealed that prenatal FOC is not only an important predictor of higher levels of postpartum FOC, but also increasing the likelihood of severe postpartum FOC. Related study has also pointed out that prenatal FOC is a risk factor for a bad childbirth experience, and the greater the fear, the higher the risk of a bad childbirth experience [25]. This finding suggests that prenatal FOC may persist into the postpartum period and impact maternal psychological status. In rural areas, rural women may not have access to adequate prenatal and postpartum care due to the relative inadequacy or unbalanced distribution of health care resources, which may not only lead to dissatisfaction with the postpartum childbirth process but also further exacerbate the degree of postpartum FOC. Therefore, by improving the accessibility and quality of health care services and implementing effective psychological support measures, it is expected to reduce women’s prenatal FOC, increase their satisfaction with childbirth, and thus improve their postpartum psychological status and promote maternal and child health.

### Study strengths and limitations

Rural women are a neglected and vulnerable group in maternal health care, and this study was the first to investigate prenatal and postnatal FOC status and its predictors among Chinese women using a prospective correlational design. However, there are limitations to consider. First, convenience sampling may introduce selection bias. Second, the study samples were obtained from a single hospital, limiting their representativeness. Second, variables related to childbirth other than mode of childbirth were not collected and analyzed in this study, which may limit the generalization of the study. Future studies should use better sampling designs, larger sample sizes, and multicenter studies to enhance the generalizability of the findings. Additionally, future research could utilize more objective and diverse measures of FOC, such as physiological and behavioral indicators. Further researches are necessary to investigate the mechanisms and regulatory factors of FOC in women, as well as effective intervention strategies to improve FOC.

## Conclusion

FOC is high among rural women in China, with approximately one in five women experiencing severe FOC both before and after childbirth. Higher education level, nullipara, higher monthly household income, lower family support, and lower childbirth self-efficacy are predictors of heightened prenatal FOC. Unemployed status, lower childbirth self-efficacy, and higher prenatal FOC are predictors of heightened postpartum FOC. Notably, enhancing childbirth self-efficacy emerges as crucial in mitigating severe prenatal FOC, while severe prenatal FOC significantly increases the likelihood of severe postpartum FOC. In the future, there is potential for a deeper exploration of the dynamic changes in FOC and the development of more tailored intervention strategies targeting the aforementioned factors. This could help alleviate women’s FOC and enhance their overall pregnancy and childbirth experience.

### Electronic supplementary material

Below is the link to the electronic supplementary material.

Supplementary Material 1

## Data Availability

The datasets that support the conclusions of this study are all available in the article.

## Abbreviations

FOC: Fear of Childbirth
WDEQ: Wijma Delivery Expectancy/ Experience Questionnaire
WDEQ-A: Wijma Delivery Expectancy/Experience Questionnaire version A
WDEQ-B: Wijma Delivery Expectancy/Experience Questionnaire version B
CBSEI: Childbirth Self-Efficacy Inventory
CBSEI-C32: 32-item Chinese Childbirth Self-Efficacy Inventory
SD: Standard Deviation
