# Supplementary material for: Pre- and postpartum fear of childbirth and its predictors among rural women in China
Source: BMC Pregnancy Childbirth. 2024 May 30;24:394. doi: 10.1186/s12884-024-06585-x (PMC11138059; doi:10.1186/s12884-024-06585-x)
Supplement: Supplementary file 1 — Supplementary Material 1 [file 12884_2024_6585_MOESM1_ESM.docx]

| **Supplementary Table 1** Fear of childbirth in pre-and postpartum periods (N=477) | | | |  |
| --- | --- | --- | --- | --- |
|  | Prenatal period  n (%)/Mean (SD) | Postpartum period  n (%)/Mean (SD) | P |  |
| Fear of childbirth, | 64.5 (25.1) | 64.3 (23.9) | 0.808 ^a^ |  |
|  |  |  | **<0.001** ^b^ |  |
| Severity of fear of childbirth |  |  | 0.247 ^c^ |  |
| Low (≤37) | 75 (15.7) | 73 (15.3) |  |  |
| Moderate (38~65) | 139 (29.1) | 155 (32.5) |  |  |
| High (66~84) | 164 (34.4) | 162 (34.0) |  |  |
| Severe (≥85) | 99 (20.8) | 87 (18.2) |  |  |
| ^a^ paired t-test; ^b^ Pearson correlation analysis; ^c^ Wilcoxon rank-sum test | | | |  |
